# Evaluation of the Analgesic Efficacy of Surgically Assisted Linea Semilunaris Block for Post-operative Analgesia in Patients Undergoing Caesarean Section Under Spinal Anaesthesia

**DOI:** 10.7759/cureus.43900

**Published:** 2023-08-22

**Authors:** Jitendra Singh, Suman Saini, Swati Bhau, Anju Gupta

**Affiliations:** 1 Department of Anaesthesiology and Critical Care, Vardhman Mahavir Medical College (VMMC) and Safdarjung Hospital, New Delhi, IND; 2 Department of Anaesthesiology, Pain Medicine, and Critical Care, AIl India Institute of Medical Sciences, New Delhi, IND

**Keywords:** rescue analgesia, spinal, abdominal wall block, regional anesthesiology, cesarean section (cs), peri-operative analgesia

## Abstract

Background: Post-operative pain following a caesarean section has been described as moderate to severe. If left untreated, the pain has a negative impact on maternal recovery and psychology. Surgically assisted linea semilunaris anterior abdominal block has been proposed to be an efficacious analgesic modality in such cases.

Aim: The study aims to evaluate the efficacy of post-operative analgesia provided by linea semilunaris block in patients undergoing caesarean section under spinal anaesthesia.

Methods: Eighty parturients planned for elective caesarean section under spinal anaesthesia were randomised into two groups. In group B, a surgically assisted Linea semilunaris anterior abdominal block was given bilaterally after the closure of the uterine incision using 20 mL of 0.375% ropivacaine with 1:200,000 adrenaline. For group C, conventional analgesia protocols were followed in the post-op period. Inj. paracetamol 1 g i.v. was routinely administered, and inj. tramadol 50 mg i.v. was given as a rescue analgesic in both groups. The primary outcome of the present study was the total amount of rescue analgesia consumed over 24 hours. Secondary outcomes included resting and dynamic pain scores [Numerical Rating Scale (NRS)], time to first rescue analgesia, quality of sleep, and patient satisfaction using the Likert scale.

Results: The mean total amount of rescue analgesia consumed over 24 hours was significantly higher in group C (150.00 ± 0.00) than in group B (125.75 ± 25.32); p = 0.001. The mean NRS at 2, 4, 12, and 24 hours was significantly higher in group B than in group C. The time to first rescue analgesia was longer in group B, with better sleep quality, patient satisfaction, and fewer complications.

Conclusion: The linea semilunaris block provided effective analgesia and can be considered an alternative analgesic modality to other conventional abdominal wall blocks for post-caesarean pain relief.

## Introduction

A caesarean section is one of the most commonly performed surgical procedures globally. Conventionally, it is performed via an infra-umbilical transverse or Pfannenstiel incision to ease delivery of the foetus [1,2]. Post-operative pain following a caesarean section, if left untreated, can have a negative impact on the maternal recovery profile and psychology. Adequate post-operative analgesia allows early ambulation, decreases thromboembolic complications, and facilitates maternal and foetal bonding. Control of pain arising post-caesarean section can be challenging as it has multifaceted components like somatosensory, visceral, and muscular components due to intraoperative repeated traction. The psychological effects of severe post-caesarean pain should also not be overlooked, as they may be associated with an adverse, fatal maternal outcome. Pain is a multi-factorial and subjective experience influenced by cultural factors, previous experiences of painful events, beliefs, and coping abilities. The patients’ personality traits can affect their level of pain perception and their response to analgesic medications. Prompt and adequate post-operative pain relief can improve maternal satisfaction and make the peripartum period more emotionally gratifying. No single optimal analgesic technique exists yet [1].

Different modalities of post-operative analgesia include systemic drugs like opioids and NSAIDs (non-steroidal anti-inflammatory drugs), neuraxial blocks, wound infiltration with local anaesthetics (LA), and peripheral nerve blocks (PNB). Among the peripheral nerve blocks, anterior and posterior abdominal wall blocks are being increasingly used nowadays [2]. Abdominal wall blocks avoid the risks and adverse effects of the central neuraxial blockade and provide a relatively safe alternative to thoracic epidural analgesia, especially where enhanced recovery protocols are used [3].

Commonly performed anterior abdominal wall blocks include the transversus abdominis plane (TAP) block, the rectus sheath (RS) block, the ilioinguinal nerve block (ILN), and the iliohypogastric nerve block (INH). The quadratus lumborum (QL) block is another upcoming posterior interfascial plane block used for caesarean patients. These blocks can be given with general anaesthesia or regional anaesthesia. PNBs given with subarachnoid blocks further prolong the duration of analgesia.

Anterior truncal blocks include a blockade of spinal nerves T7-L1 running in the interfacial plane between the internal oblique and transversus abdominis muscle, i.e., the TAP. These myocutaneous nerves pierce the rectus sheath at linea semilunaris (LS) and become superficial as terminal anterior and lateral cutaneous branches pass towards linea alba. They supply the parietal peritoneum, but not the muscles and skin of the anterior abdominal wall [2].

Various methods of performing anterior abdominal wall blocks are blind, ultrasound guided (USG), or surgically guided. Blind blocks have the advantages of simplicity in learning and performing, a faster block procedure, and minimal requirements for the equipment. However, they can be associated with complications like block failure, intravascular injection, or injection into the peritoneal cavity [4].

Over the past decade, ultrasound-guided blocks have gained popularity over blind blocks as they allow real-time visualisation of the needle and the injected drug, making the block more accurate and safer. However, technical difficulties have been reported with USG-guided blocks in obstetric patients due to a gravid uterus, difficult positioning, excessive subcutaneous adipose tissue, and a greater risk of bleeding due to engorged vessels. Also, there is a limitation of drug spread in patients with high BMI (body mass index) due to decreased potential space between the RS and transversalis fascia at the level of the navel in the rectus sheath block (RSB) [5].

Recently, a newer regional anaesthesia technique, the 'linea semilunaris block' has been described by Akade et al. in caesarean patients, which targets bilateral injection of local anaesthetics into the anterior RS at the linea semilunaris below the arcuate line. The drug is injected in the myofascial plane between the tendons of the anterior abdominal muscles and the fascia transversalis [1]. The RSB aims to block the terminal branches of the 9th, 10th, and 11th intercostal nerves located in the space between the rectus abdominis muscle and its posterior RS [6]. It has been used for postoperative analgesia extending along the midline for upper abdominal surgeries, abdominal gynaecological procedures, and abdominoplasty [7-9].

However, an extensive spread of LA has been reported by authors with the linea semilunaris block, not only in the TAP plane but also along the fascia transversalis going posteriorly towards the quadratus lumborum (QL) muscle, as observed in cadaveric dye studies [10]. The linea semilunaris block is a technique that combines the analgesic efficacy of both the TAP block and, to some extent, the QL block. We, therefore, aimed to explore its analgesic efficacy in patients undergoing caesarean section with the primary aim of comparing analgesic consumption in the two groups. The secondary aim was to study the pain scores using the Numeric Rating Scale (NRS), sleep quality, patient satisfaction, and complications in the two groups.

## Materials and methods

This was a prospective randomised clinical study conducted in a tertiary care hospital after prior approval from the institutional ethics committee and registration in the clinical trial registry of India. Written and informed consent was obtained from all the patients after explaining the objectives of the study, the technique, and its related complications.

Eighty healthy term pregnant females belonging to American Society of Anesthesiologists (ASA) physical status II who were planned for elective caesarean section under spinal anaesthesia were randomised into two groups according to the block randomization technique (in blocks of 10) using computer-generated random numbers maintained in sequentially numbered sealed envelopes for allocation concealment. In group B (n=40), a surgically assisted linea semilunaris block was given bilaterally after the closure of the uterine incision using 20 mL of 0.35% ropivacaine with 1:200,000 adrenaline. For group C (n=40), conventional analgesia protocols were followed in the post-operative period. Patient blinding was not possible due to the study design, as the block was performed in only one group. However, the post-operative outcome assessor who recorded the analgesic outcomes was blinded to the study groups.

Sample size

The study population was calculated using G-power software with 80% power and a 5% significance level. A previous study observed that cumulative tramadol usage during the first 24 hours after surgery in the control group was 168 ± 45 mg [1]. Taking these values as a reference and assuming a difference of 25% in cumulative tramadol usage between linea semilunaris anterior abdominal block in comparison to conventional analgesia after subarachnoid block, the minimum required sample size with 80% power of the study and a 5% level of significance is 30 patients in each study group. To reduce the margin of error, the total sample size taken was 80 (40 patients per group).

All patients underwent a detailed pre-anaesthesia check-up and were kept fasting after midnight. The procedure and the advantages and side effects of both techniques were explained to the patients, and they were also explained the use of an 11-point NRS scale (0-10), where '0' means no pain at all and '10' means the worst pain experienced. All patients were administered pantoprazole 40 mg orally at night and two hours prior to surgery.

The patients were shifted to the operating theatre, and standard monitors, including electrocardiography (ECG), non-invasive blood pressure (NIBP), and pulse oximeter (SpO_2_), were applied. Baseline heart rate (HR), mean arterial pressure (MAP), SpO_2_, and respiratory rate (RR) were noted. An intravenous (IV) access was secured using a 20G cannula on a non-dominant hand, and intravenous fluid (Ringers Lactate) was started. The patients were informed about the procedure. Each patient was given subarachnoid block spinal anaesthesia with 11 mg (2.2 mL) of 0.5% heavy bupivacaine at L3-L5 level in a sitting position after preloading 10 mL/kg of Ringers lactate. An adequate level of sensory and motor anaesthesia was ensured and noted. Oxygen at a flow rate of 5 L/min was given via a Hudson mask. Vital monitoring was continued throughout the perioperative period. After completion of the surgery, the patients in group C were immediately shifted to the post-anaesthesia care unit (PACU), and monitoring was continued. In group B, the patients received a surgeon-assisted Linea semilunaris block following the uterine closure prior to the closure of the abdominal wall, as described below [1]. Following the block procedure, the abdomen was closed, and patients were shifted to the PACU for further monitoring.

Linea semilunaris block procedure

**Figure 1:**
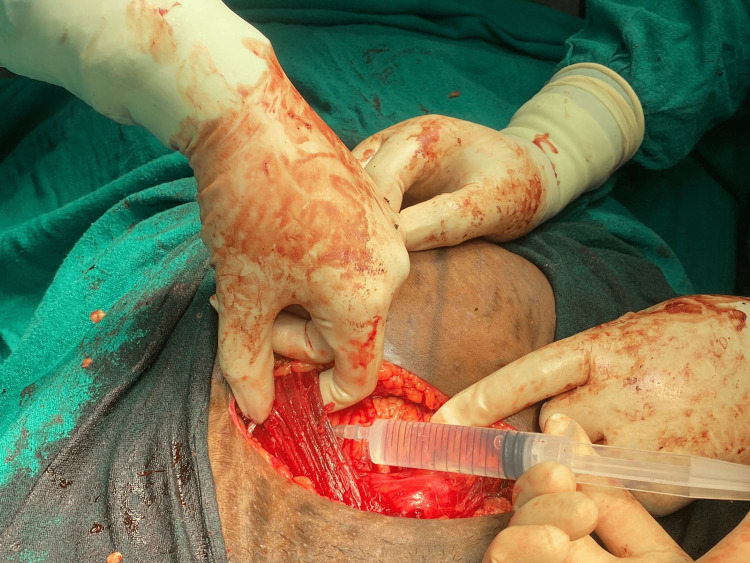
Block procedure (a) Injection of local anaesthetic by the anaesthetist after surgical dissection of the plane between linea semilunaris and fascia transversalis (1b).] Adapted from Akhade et al. [1] (this article is available under the terms of the Creative Commons Attribution-Non-Commercial-Share-Alike License (CC BY-NC-SA), which permits non-commercial use, distribution and reproduction in any medium, provided the original work is properly cited).

After the closure of the uterine incision, the RS was retracted cranially by the surgeon. Under all aseptic precautions, the block was given by an anesthesiologist using a 22G hypodermic needle attached to the 10-mL syringe at two sites. A needle was inserted at the linea semilunaris in the anterior RS below the arcuate line from the medial to the lateral direction. The local anaesthetic injection was in the myofascial plane, between the tendons of the anterior abdominal muscles above and the fascia transversalis and peritoneum below. First, the needle was directed towards the petit triangle of the iliac region at 10° to 20° angulation. Then it was redirected towards the lumbar region at the 3 o'clock position (for the left side), and a total volume of 20 mL of 0.375% ropivacaine with 1:200,000 adrenaline was administered after careful negative aspiration. While injecting the drug, the anaesthesiologist kept his/her other hand below the rectus muscle and peritoneum to avoid any bowel injuries. The same volume of local anaesthetic was injected on another side of the anterior abdominal wall following a similar technique before closing the surgical wound (Figure 1).

Parameters monitored

Intra-operatively, HR, MAP, and SpO_2_ were noted every five minutes for the first 15 minutes after administering spinal anaesthesia and then every 10 minutes until the completion of surgery.

Post-operatively, the total amount of rescue analgesia required in each group over 24 hours and the time of first rescue analgesia were noted. NRS at rest was noted at 0 (at the time of shifting the patient to the PACU), 2, 4, 8, 12, and 24 hours from the completion of surgery. Dynamic NRS was noted at the same time points by passively raising legs. Complications, if any, related to the procedure, like pain, vascular puncture, and signs of local anaesthetic toxicity, were noted. Side effects of analgesics like nausea, vomiting, sedation, pruritis, and urinary retention were monitored for the first 24 hours. Quality of sleep and patient satisfaction in terms of analgesia were noted at 24 hours using the Likert scale (very unsatisfied, unsatisfied, neutral, satisfied, very satisfied).

Post-operatively, all patients were administered inj. paracetamol (1 g) and inj. ondansetron (4 mg) by eight-hourly IV. For breakthrough pain, rescue analgesia in the form of inj. tramadol 1 mg/kg was administered IV over 20-30 min on demand or if NRS score >4 and its time was noted.

Statistical analysis

The data were charted in Microsoft Excel (Microsoft® Corp., Redmond, WA), and the statistical analysis was performed by statistical software SPSS version 21.0 (IBM Corp., Armonk, NY). The quantitative (numerical variables) were presented in the form of mean and standard deviation (SD), and the qualitative (categorical variables) were presented in the form of frequency and percentage.

The student t-test was used for comparing the mean values between the two groups, whereas the chi-square test was applied for comparing the frequency. The p-value was considered significant when it was less than 0.05.

## Results

The efficacy of linea semilunaris block was studied in 80 ASA II parturients (pregnant females with no comorbidity) undergoing Caesarean surgery from September 2020 to July 2021. Ninety patients posted for caesarean section in the study period were assessed for eligibility, out of which six patients were excluded based on exclusion criteria, four patients were denied permission to be included in the study, and finally, 80 patients were recruited. In the two groups B and C, the patients enrolled in this study were compared with respect to the demographic profile, and no statistically significant difference was found between the two groups (Table 1).

**Table 1:** Demographic parameters of the patients in the two groups BMI: body mass index

Parameters	Group B	Group C	P-value
Age (years)	27.50±6.45	29.20±5.69	0.215
Weight (kg)	58.60±2.38	60.93±5.70	0.120
Height (m)	1.64±0.11	1.65±0.12	0.595
BMI (kg/m^2^)	22.14±2.96	22.55±2.91	0.526
Duration of surgery (min)	62.58±9.18	62.68±9.54	0.960

The mean NRS score at 2 hours, 4 hours, 12 hours, and 24 hours was significantly higher in group C (2.40±0.50, 2.68±0.47, 4.93±0.73, and 4.70±0.52, respectively) compared to group B (1.60±0.59, 3.89±0.47, 4.01±0.82, and 4.03±0.53, respectively) (Table 2).

**Table 2:** Comparison of post-operative NRS at rest, dynamic NRS, and time to first recue analgesia in the two groups

	Group B Mean(SD)	Group C Mean(SD)	P value
NRS at rest			
0 hour	0.25(0.63)	0.13(0.34)	0.272
2 hours	1.60(0.59)	2.40(0.50)	0.001*
4 hours	2.25(0.50)	3.89(0.47)	0.003*
6 hours	3.60(0.81)	3.94(0.80)	0.137
12 hours	4.01(0.82)	4.93(0.73)	0.011*
24 hours	4.03(0.53)	4.70(0.52)	0.001*
NRS on movement			
0 hour	0.25(0.63)	0.25(0.44)	1.000
2 hours	1.65(0.66)	2.75(0.44)	0.001*
4 hours	3.43(0.50)	3.98(0.49)	0.043*
6 hours	4.60(0.81)	4.93(0.80)	0.132
12 hours	5.18(0.82)	5.93(0.73)	0.011*
24 hours	5.03(0.53)	5.70(0.52)	0.001*
Time to first recue analgesia (hours)	9.13(2.71)	5.61(0.43)	0.001
Total rescue analgesic required (mg)	150(0)	125(25.3)	0.001
Need for recue analgesia (n)			
2 hours	0	0	1
4 hours	0	11	0.00
6 hours	18	30	0.001
12 hours	4	40	0.040
24 hours	38	40	0.91

The mean NRS (dynamic) at 2 hours, 4 hours, 12 hours, and 24 hours was significantly higher among group C (2.75±0.44, 3.98±0.49, 5.93±0.73, and 5.70±0.52, respectively) compared to group B (1.65±0.66, 3.43±0.50, 5.18±0.82, and 5.03±0.53, respectively) (Table 2).

The mean time to first rescue analgesia was significantly longer in group C (9.13±2.71 hours) compared to group B (5.61±0.43 hours; p-value < 0.05). The mean dose of rescue analgesia required at 4, 6, and 12 hours was significantly higher in group C (3.75±13.34 mg, 46.25±13.34 mg, and 50.00±0.00 mg, respectively) compared to group B (0.00±0.00, 22.00±25.32, respectively); p-value < 0.05. The mean total rescue analgesia required in 24 hours was significantly (p-value<0.05) higher among group C (150.00±0.00 mg) compared to group B (125.75±25.32 mg); p= 0.001 (Table 2). Nausea and vomiting were higher in group C as compared to group B.

## Discussion

There has been a dramatic rise in CS in the past two decades, making it the most commonly performed surgical procedure worldwide [11]. Although advances have been made in understanding the pathophysiology of post-caesarean pain and the development of new analgesics and delivery techniques, many patients still suffer from moderate-to-severe pain [12]. The analgesic regimen should provide safe, effective analgesia with minimal side effects for the mother and baby.

As a part of the multimodal analgesia regime, opioids used via intravenous, epidural, and intrathecal routes are associated with undesirable side effects and create an opportunity for truncal blocks. Pain after CS is challenging and has three components: somatosensory pain originating from the cutaneous, subcutaneous, and muscular layers of the incision site; visceroperitoneal inflammatory pain of the viscera and deeper peritoneal layers; and psychosomatic pain [13].

The present prospective randomised clinical study evaluated the efficacy of a surgically assisted linea semilunaris anterior abdominal wall block for post-operative analgesia in patients undergoing caesarean section under spinal anaesthesia. In our study, the mean NRS score at rest at 0 hours was between groups B and C, respectively, which did not differ significantly because the analgesia provided by the subarachnoid block was present until that time. However, at two and four hours, the mean NRS was significantly higher in group C compared to group B. At six hours, the NRS between groups B and C did not differ significantly, as rescue analgesia was given to some of the patients at four hours in group C, and consequently, the NRS score was reduced at six hours between both groups. As the effects of the anaesthesia and analgesia wore off, the mean NRS at 12 and 24 hours was significantly higher in group C compared to group B. Dynamic NRS was noted by raising legs at the same time intervals. Similar trends were observed in the dynamic NRS at different time points. Our results are in concurrence with Akhade et al., who also conducted a study to evaluate the post-operative analgesic efficacy of surgically assisted abdominal field block at linea semilunaris after the closure of the uterine incision by using 20 mL of 0.25% bupivacaine on each side in 120 parturients undergoing caesarean section [1]. None of the patients in the study group experienced severe or worse pain. Similarly, Abo-Zeid et al. [14] demonstrated that post-abdominoplasty analgesic duration consequent to the three surgically infiltrated local anaesthetic techniques: bilateral TAPB, bilateral RSB, and subcutaneous infiltration (SCI) of 0.25% bupivacaine, was greatest with TAP block compared with both the RS block and SCI groups. Statistically significant higher VAS scores in the SCI group four hours post-operatively were recorded, both at rest and during movement, compared with both the TAP block and RS block groups. Significantly higher morphine consumption was found in the SCI group compared with the other two groups. Since linea semilunaris block is similar to TAP block in mechanism, with an additional element of spread to the quadratus lumborum plane, it can be concluded that it would be better than RS block in analgesic efficacy.

In our study, the mean time to first rescue analgesia was significantly (p-value < 0.05) longer in group C compared to group B. As the pain control lasted for a longer duration among the block group, which can be attributed to the addition of adrenaline along with ropivacaine, the first analgesia was given at a later interval in group B in comparison to group C. Similar results were observed in a previous study on this block [1]. Jadon et al. [15] evaluated the analgesic efficacy of TAP block for post-caesarean analgesia in a randomised controlled trial. The median (interquartile range) time to the first analgesic request was prolonged in the TAP group compared to the control group: 11 hours (8,12) and 4 hours (2.5, 6), respectively. The median (interquartile range) number of doses of tramadol consumed in the TAP group was 0 (0,1) compared to 2 (1,2) in the control group. At all points in the study, pain scores both at rest and during movement were lower in the study group. Maternal satisfaction with pain relief was also higher in the study group.

There has been a dramatic rise in CS in the past two decades, making it the most commonly performed surgical procedure worldwide [11]. Although advances have been made in understanding the pathophysiology of post-caesarean pain and in the development of new analgesics and delivery techniques, many patients still suffer from moderate-to-severe pain [12]. The analgesic regimen should provide safe, effective analgesia with minimal side effects for the mother and baby.

As a part of the multimodal analgesia regime, opioids used via intravenous, epidural, and intrathecal routes are associated with undesirable side effects and create an opportunity for truncal blocks. Pain after CS is challenging and has three components: somatosensory pain originating from the cutaneous, subcutaneous, and muscular layers of the incision site; visceroperitoneal inflammatory pain of the viscera and deeper peritoneal layers; and psychosomatic pain [13].

The present prospective randomised clinical study evaluated the efficacy of surgically assisted linea semilunaris anterior abdominal wall block for post-operative analgesia in patients undergoing caesarean section under spinal anaesthesia. In our study, the mean NRS score at rest at 0 hours was between groups B and C, respectively, which did not differ significantly because the analgesia provided by the subarachnoid block was present until that time. However, at two and four hours, the mean NRS was significantly higher in group C compared to group B. At six hours, the NRS scores among groups B and C did not differ significantly, as rescue analgesia was given to some of the patients at four hours in group C, and consequently, the NRS score was reduced at six hours among both groups. As the effects of the anaesthesia and analgesia wore off, the mean NRS at 12 and 24 hours was significantly higher in group C compared to group B. Dynamic NRS was noted by raising legs at the same time intervals. Similar trends were observed in the dynamic NRS at different time points. Our results are in concurrence with Akhade et al., who also conducted a study to evaluate the post-operative analgesic efficacy of surgically assisted abdominal field block at linea semilunaris after the closure of the uterine incision by using 20 mL of 0.25% bupivacaine on each side in 120 parturients undergoing caesarean section [1]. None of the patients in the study group experienced severe or worse pain. Similarly, Abo-Zeid et al. [14] demonstrated that post-abdominoplasty analgesic duration consequent to the three surgically infiltrated local anaesthetic techniques: bilateral TAPB, bilateral RSB, and SCI of 0.25% bupivacaine, was greatest with TAP block compared with both the RS block and SCI groups. Statistically significant higher VAS scores in the SCI group four hours post-operatively were recorded, both at rest and during movement, compared with both the TAP block and RS block groups. Significantly higher morphine consumption was found in the SCI group compared with the other two groups. Since linea semilunaris block is similar to TAP block in mechanism, with an additional element of spread to the quadratus lumborum plane, it can be concluded that it would be better than RS block in analgesic efficacy.

In our study, the mean time to first rescue analgesia was significantly (p-value < 0.05) longer in group C compared to group B. As the pain control lasted for a longer duration among the block group, which can be attributed to the addition of adrenaline along with ropivacaine, the first analgesia was given at a later interval in group B in comparison to group C. Similar results were observed in a previous study on this block [1]. Jadon et al. [15] evaluated the analgesic efficacy of TAP block for post-caesarean analgesia in a randomised controlled trial. The median (interquartile range) time to the first analgesic request was prolonged in the TAP group compared to the control group: 11 hours (8, 12) and 4 hours (2.5, 6), respectively. The median (interquartile range) number of doses of tramadol consumed in the TAP group was 0 (0,1) compared to 2 (1,2) in the control group. At all points in the study, pain scores both at rest and during movement were lower in the study group. Maternal satisfaction with pain relief was also higher in the study group.

In the present study, the mean dose of rescue analgesia (tramadol) required was at 4, 6, and 12 hours and was significantly higher in group C compared to group B. The mean total rescue analgesia required was significantly higher in group C compared to group B. As the block was more effective in pain control in the immediate post-operative period and the requirement for additional analgesia was lesser in group B as compared to group C. This is in agreement with the study by Akhade et al. [1], where mean analgesic consumption over a 24-hour post-caesarean section was significantly less in the study group. No patient required opioid supplementation. Siddiqui et al. [16] and Charlton et al. [17] conducted a meta-analysis to review the clinical effectiveness of anterior abdominal wall block (TAP block). They both demonstrated a morphine-sparing effect of TAP blocks in the first 24 hours after a caesarean section, thereby reducing opioid-related side effects.

Quality of sleep and patient satisfaction assessed in terms of analgesia using the Likert scale were significantly higher in group B compared to group C. Previous studies had also reported higher patient satisfaction in the linea semilunaris block group compared to the control group [1].

Nausea and vomiting were significantly more common in group C compared to group B. Baaj et al. [18] compared bilateral TAP block for post-op analgesia with either local anaesthetic or saline and found a significant reduction in 24-hour morphine requirement in the study group versus controls (26±5 mg vs. 63±5 mg). The authors also reported lower 24-hour VAS scores, lower PONV scores, and higher satisfaction in the study group compared to the placebo group.

Our study has certain limitations. It was a single-centre study; further multicentric trials can validate the results. Due to the nature of the intervention, which was performed in one group only, the patient and the anaesthesiologist who performed the block could not be blinded. However, the outcomes were assessed post-operatively by an independent anaesthesiologist who was not involved in intraoperative care, thus minimising the possibility of any bias. The use of a sham block in the control group could have removed the possibility of patient bias, further lending strength to the study design. Further, due to access and skill with ultrasound-guided blocks, this block may have limited application in the future. However, considering its effectiveness and quick block procedure, the block merits further investigation.

## Conclusions

Analgesia for the obstetric patient is challenging as it affects both the foetus and the mother. There is a lot of potential for the linea semilunaris block to comprise an effective component of a multimodal regimen for post-caesarean section analgesia, and it is also easy to perform. It can be recommended that the Linea semilunaris block is more accurate because it is under vision, simple and easy to perform, opioid-sparing, and provides high patient satisfaction. The block should be considered as an alternative to other blocks for the post-op relief of pain in a patient undergoing a caesarean section.
